# My anesthesia Choice-HF: development and preliminary testing of a tool to facilitate conversations about anesthesia for hip fracture surgery

**DOI:** 10.1186/s12871-024-02547-0

**Published:** 2024-05-01

**Authors:** Mark D. Neuman, Glyn Elwyn, Veena Graff, Viktoria Schmitz, Mary C. Politi

**Affiliations:** 1 Department of Anesthesiology and Critical Care, University of Pennsylvania Perelman School of Medicine, 308 Blockley Hall 423 Guardian Drive, Philadelphia, PA 19106 USA; 2 Leonard Davis Institute of Health Economics, University of Pennsylvania, Philadelphia, USA; 3 Center for Perioperative Outcomes Research and Transformation, University of Pennsylvania Perelman School of Medicine, Philadelphia, USA; 4 Department of Medicine, Division of Geriatric Medicine, University of Pennsylvania Perelman School of Medicine, Philadelphia, USA; 5 Dartmouth College, Hanover, NH USA; 6 Division of Public Health Sciences, Department of Surgery, Washington University in St. Louis, St. Louis, USA

**Keywords:** Hip fractures, General and spinal anesthesia, Patient-centered outcomes, Shared decision-making, Conversation aid

## Abstract

**Background:**

Patients often desire involvement in anesthesia decisions, yet clinicians rarely explain anesthesia options or elicit preferences. We developed My Anesthesia Choice-Hip Fracture, a conversation aid about anesthesia options for hip fracture surgery and tested its preliminary efficacy and acceptability.

**Methods:**

We developed a 1-page, tabular format, plain-language conversation aid with feedback from anesthesiologists, decision scientists, and community advisors. We conducted an online survey of English-speaking adults aged 50 and older. Participants imagined choosing between spinal and general anesthesia for hip fracture surgery. Before and after viewing the aid, participants answered a series of questions regarding key outcomes, including decisional conflict, knowledge about anesthesia options, and acceptability of the aid.

**Results:**

Of 364/409 valid respondents, mean age was 64 (SD 8.9) and 59% were female. The proportion indicating decisional conflict decreased after reviewing the aid (63–34%, *P* < 0.001). Median knowledge scores increased from 50% correct to 67% correct (*P* < 0.001). 83% agreed that the aid would help them discuss options and preferences. 76.4% would approve of doctors using it.

**Conclusion:**

My Anesthesia Choice-Hip Fracture decreased decisional conflict and increased knowledge about anesthesia choices for hip fracture surgery. Respondents assessed it as acceptable for use in clinical settings.

**Practice implications:**

Use of clinical decision aids may increase shared decision-making; further testing is warranted.

**Supplementary Information:**

The online version contains supplementary material available at 10.1186/s12871-024-02547-0.

## Background

Over 1.6 million adults worldwide undergo surgery to treat hip fractures each year, most often with spinal anesthesia (numbing the lower extremities) or general anesthesia (medically induced unconsciousness) [1]. Both anesthesia options are safe and effective for keeping patients comfortable during surgery. Recent large randomized trials comparing the effects of spinal versus general anesthesia on patient-centered outcomes have shown that recovery of walking, delirium, length of stay, pain, and satisfaction were similar with either option [2–4]. The decision about which anesthesia to use during hip fracture surgery is therefore preference-sensitive, depending on patients’ understanding of and preferences for aspects of different anesthetic techniques (e.g., spinal injection, endotracheal intubation) as well as clinical contraindications for either option.

Shared decision-making between patients and clinicians is recommended when there are choices that should be informed by both clinical evidence and patients’ preferences. Brief conversation aids can facilitate shared decision-making in contexts in which patients may not have had time to prepare in advance for a decision discussion, but still have time to discuss options with clinicians prior to treatment [5–7]. While many patients want to be involved in anesthesia care decisions [8], anesthesia teams rarely explain risks and benefits of options and frequently fail to elicit or integrate patient preferences [9]. Little is known about the effectiveness and acceptability of conversation aids to guide decisions about anesthesia care for hip fracture.

## Objective

**Fig. 1:**
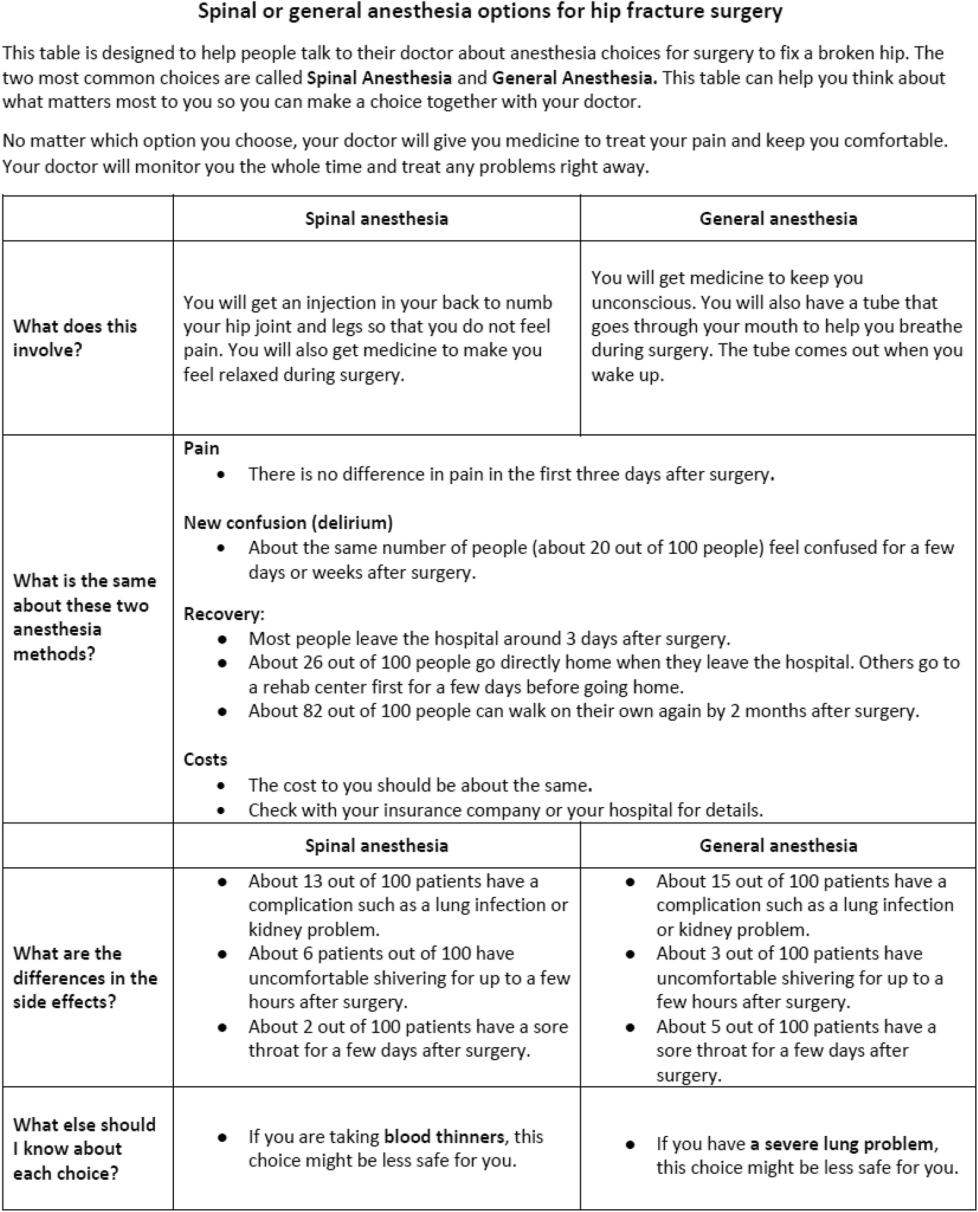
My anesthesia Choice-Hip fracture conversation aid

The study authors drafted My Anesthesia Choice-HF, a 1-page conversation aid about anesthesia options for hip fracture surgery, using outcomes data drawn from a multicenter randomized trial comparing spinal versus general anesthesia for hip fracture surgery [2, 3]. The aid was refined based on informal feedback from experts with expertise in orthopedic and regional anesthesia and shared decision making, a 5-member community advisory panel, and 5 additional members of the public. Authors reviewed feedback obtained from these assessments and decided on revisions to the tool based on consensus. Edits were made to content, format and language to ensure it was clear, understandable and accurate. In developing the tool, we adopted the format of an Option Grid™ conversation aid. Option Grids [5–7] are short tabular comparisons of options, incorporating best available evidence that can be reviewed by patients and clinicians together at the bedside to facilitate efficient conversations during visits and promote deliberation about choices [5, 6, 10–12]. Option Grids, have been shown to improve the quality of conversations about treatment options between patients and clinicians in a range of treatment settings [5]. Our grid compared spinal and general anesthesia and was organized as responses to a series of common questions, written at a 7th grade reading level (Fig. 1).

The objective of this study was to test the preliminary efficacy and acceptability of My Anesthesia Choice-HF in an online experiment prior to using or testing its effectiveness in clinical contexts. We hypothesized that use of the aid would be associated with increased knowledge and decreased decisional conflict when considering a hypothetical choice between spinal and general anesthesia.

## Methods

We tested the preliminary efficacy and acceptability of My Anesthesia Choice-HF in a sample recruited from Prime Panels (Prime Research Solutions, Flushing, NY), a compilation of online survey panels that reaches over 100 million individuals worldwide [13, 14]. We selected Prime Panels as the platform for survey administration because the participant pool is diverse and resembles the demographic patterns of the general US adult population, [13]. Eligible participants were English-speaking adults aged 50 and older. The study approval was waived by the institutional review board (IRB) of the Washington University in St. Louis IRB.

Participants read a hypothetical scenario (see Additional file 1) in which they were asked to imagine having to choose between spinal and general anesthesia for hip fracture surgery. Participants were informed that “for many patients, either of these options may be safe, but there are still differences patients might want to consider.” Participants then completed the 4-item SURE assessment for decisional conflict (Sure of myself; Understand information; Risk-benefit ratio; Encouragement) [15], and 6 items assessing knowledge about anesthesia options. Participants next reviewed the My Anesthesia Choice-HF aid in PDF format; after viewing the aid, we re-administered all knowledge items and the SURE assessment. Additional survey items assessed acceptability of the aid, willingness to use it in conversations with physicians about actual anesthesia choices, and sociodemographic characteristics.

Following exclusion of respondents with survey completion time < 3 min, a straight-line answer pattern, or an incorrect response to an embedded attention check, we compared the number of correctly answered knowledge items for each participant before vs. after reviewing the aid using the Wilcoxon signed-rank test. We compared the proportion of individuals indicating decisional conflict before vs. after reviewing the aid using McNemar’s test. For this initial exploratory analysis, we aimed to recruit a sample of 400 participants to allow us to exclude moderate associations between instrument use and outcomes. For acceptability items, we calculated the proportion indicating agreement to a given item and its corresponding confidence interval. Analyses used Stata 16.1 (Statacorp, College Station, TX).

## Results

Survey administration occurred on 19 December 2022. Of 409 total respondents, 8 were excluded for age below 50 years and 37 were excluded based on data quality checks, leaving an analytic sample of 364 respondents (89.0%). Mean age was 63.9 (SD 8.9), 58.7% were female, and the highest education level attained was a high school diploma or less in 34.1% (Table 1).

**Table 1:** Sample characteristics and responses to acceptability items^a^

**Sample Characteristics**	**Mean (SD)**
**Age**	63.8 (8.9)^b^
**Gender**	**n/N (%)**
Male	150/363 (41.3)
Female	213/363 (58.7)
**Education**	**n/N (%)**
Less than a high school degree	15/363 (4.1)
A high school diploma	93/363 (25.6)
Technical certification	16/363 (4.4)
Some college	101/363 (27.8)
A college degree	91/363 (25.1)
Graduate/professional degree	47/363 (13.0)
**Ethnicity**	**n/N (%)**
Latin/x or hispanic	15/354 (4.2)
**Race**	**n/N (%)**
White	313/361 (86.7)
Black	31/361 (8.6)
More than one or other	17/361 (4.7)
**Income**	**n/N (%)**
$75,000 or more	86/345 (24.9)
$60,000-$74,999	29/345 (8.4)
$45,000- $59,999	54/345 (15.7)
$30,000-$44,999	51/345 (14.8)
$15,000-$29,999	92/345 (26.7)
Less than $15,000	33/345 (9.6)
**Acceptability item responses**	**Respondents indicating agreement, n/N, %, 95% CI**
I would approve of my doctor using the grid about anesthesia choices^c^	278/364, 76.4 (71.7, 80.6)
Having my doctor use the grid about anesthesia choices is appealing to me^c^	258/364, 70.9 (65.9, 75.4)
I would like my doctor to use the grid about anesthesia choices with me^c^	269/364, 73.9 (69.1, 78.3)
I would welcome my doctor using the grid about anesthesia choices^c^	272/364, 74.7 (69.9, 79.1)
The grid about anesthesia choices make your treatment decision would make treatment decision easier^d^	279/364, 76.7 (72.0, 80.9)
The grid about anesthesia choices has enough information to help someone decide on treatment options	299/362, 82.6 (78.3, 86.4)
The grid about anesthesia choices would help you talk about your choice with your doctor or other people on your care team^d^	312/364, 85.7 (81.7, 89.1)

*CI *Confidence Interval, *SD *Standard Deviation

^a^Denominators vary across items due to missing responses

^b^Data available on 354 respondents

^c^Responses measured on a 5-point Likert-type scale ranging from “strongly agree” to “strongly disagree;” a response of “agree somewhat” or “strongly agree” was considered to indicate agreement with a given item

^d^Denominator includes respondents indicating “unsure” for this item

The proportion indicating decisional conflict before versus after reviewing the aid decreased from 63.2% (134/364) to 34.3% (125/364; *P* < 0.001). The median knowledge test score at baseline was 3 of 6 items correct (50.0%; interquartile range (IQR), 2,3); median score after reviewing the My Anesthesia Choice-HF aid was 4 of 6 items correct (66.7%; IQR, 3, 5.5; *P* < 0.001). 83% of participants agreed that the aid would help them discuss these options and form views about their preferences and 76.4% would approve of their doctor using it; agreement was similarly high for other acceptability items assessed (Table 1).

## Discussion

In a national survey of 364 adults aged 50 or older, we found that reviewing a 1-page, plain-language conversation aid—the My Anesthesia Choice-HF tool—decreased decisional conflict and increased knowledge about care options related to a hypothetical choice between spinal anesthesia or general anesthesia for hip fracture surgery. Four out of 5 respondents agreed that the aid would help them discuss options and preferences, and over 3 out of every four respondents would approve of doctors using it. Strengths of this study include the stakeholder-engaged development process of the conversation aid, the sample size that was adequate to test preliminary efficacy and acceptability, and ethics of using a non-clinical sample to test the aid before using it with patients in practice.

While our study succeeded in recruiting a large national sample to assess this tool, there were several limitations. We recruited our respondents from a general adult population rather than actual patients undergoing hip surgery. First, it is possible that involving a clinical population in the study could have produced different results, particularly if time constraints or anxiety prior to surgery impacted patients’ responses, or if existing cognitive impairment influenced patients’ ability to engage with the decision aid. Second, respondents did not review the tool in the presence of an anesthesiologist, as would often be the case in typical care. It is possible that we could have obtained different results regarding the utility and acceptability of the tool had it been accompanied by counseling from a clinician.

Third, we used a hypothetical scenario as the basis for our survey; results could differ in actual hip fracture patients. Fourth, we did not collect information on why some individuals indicated that they would not approve of their doctor using the aid. Further research may explore the reasons for such attitudes among a minority of patients. Fifth, because of the large number of potentially eligible individuals for Prime Panels surveys, we were unable to compute a denominator for the study sampling frame. Finally, the generalizability of the study may be limited by the fact that panel respondents may not be fully representative of patients experiencing hip fractures. For example, the average age of patients in our sample was lower than has been reported in population-based samples of hip fracture patients [16].

Despite these limitations, our work provides initial evidence of the efficacy and acceptability of the My Anesthesia Choice-HF aid. Further refinement of the aid and evaluation in clinical contexts is needed to assess the potential for the aid to increase shared decision making and improve patient-centered outcomes regarding anesthesia choices for hip fracture surgery.

## Conclusion

Use of the My Anesthesia Choice-HF conversation aid was associated with decreased decisional conflict and increased knowledge when considering a hypothetical choice between spinal and general anesthesia for hip fracture surgery. Most respondents found the tool acceptable for clinical use. Results indicate the potential for My Anesthesia Choice-HF to be tested in a clinical setting to assess potential barriers and promoters to implementation and to assess its potential to impact clinical decisions.

### Supplementary Information

**Supplementary Material 1.**

## Data Availability

The datasets generated during and/or analyzed during the current study are available from the corresponding author on reasonable request.
