# Supplementary material for: My anesthesia Choice-HF: development and preliminary testing of a tool to facilitate conversations about anesthesia for hip fracture surgery
Source: BMC Anesthesiol. 2024 May 1;24:165. doi: 10.1186/s12871-024-02547-0 (PMC11061990; doi:10.1186/s12871-024-02547-0)
Supplement: Supplementary file 1 — Supplementary Material 1. [file 12871_2024_2547_MOESM1_ESM.pdf]

Appendix: Study Survey Instrument

First, we will ask you some questions about what you know about anesthesia and how you feel about anesthesia choices. Then, we will share some information with you about these choices and ask you the same questions again. We don't expect people to know all of this information. Our goal is to learn about what people know, and how they want to make this choice, so we can come up with ways to help patients who are facing this choice.

Imagine that you fell and broke your hip. Your doctor tells you that you need to have surgery for your hip. During the surgery, you will need anesthesia so that you do not feel pain. The two most common options for anesthesia for hip fracture surgery are called **spinal anesthesia** and **general anesthesia**. Research has shown that for many patients, either of these options may be safe, but there are still differences patients might want to consider.

Imagine that you are about to have surgery and you are asked to choose between spinal anesthesia and general anesthesia:

SURE measure of decisional conflict	**Yes**	**No**	**Unsure**
1. Would you feel sure about the best choice for you?	○	○	○
2. Would you know the benefits and risks of each option?	○	○	○
3. Would you feel clear about which benefits and risks matter most to you?	○	○	○
4. Would you have enough support and advice to make a choice?	○	○	○

Please answer true, false, or unsure to the next questions. Please tell us what you think, without looking anything up. We don't expect you to know all of these answers.

Knowledge questions	**True**	**False**	**Unsure**
5. **Spinal anesthesia** uses an injection in your back to numb your legs and hips so that you don't feel pain during surgery.	○	○	○
6. More people feel confused after **general anesthesia** than after **spinal anesthesia**.	○	○	○

Knowledge questions	True	False	Unsure
7. It takes a longer time to recover after **general anesthesia** than from **spinal anesthesia.**	○	○	○
8. No matter which type of anesthesia you choose, your doctor will give you medicine to treat your pain and keep you comfortable during surgery.	○	○	○
9. If someone has severe lung disease, that can make **general anesthesia** less safe for them.	○	○	○
10. If someone take blood thinners, that can make **general anesthesia** less safe for them.	○	○	○

11. Did you look up the answers? Please answer honestly.

Yes

No

Thank you. Now we would like to show you some information about general and spinal anesthesia.

(*Note: My Anesthesia Choice-HF Grid presented here*)

Please answer true, false, or unsure to the next questions. For these, you can refer back to the information we showed you in the grid if you are not sure.

	True	False	Unsure
12. **Spinal anesthesia** uses an injection in your back to numb your legs and hips so that you don't feel pain during surgery.	○	○	○
13. More people feel confused after **general anesthesia** than after **spinal anesthesia**.	○	○	○
15. It takes a longer time to recover after **general anesthesia** than from **spinal anesthesia.**	○	○	○
16. No matter which type of anesthesia you choose, your doctor will give you medicine to treat your pain and keep you comfortable during surgery.	○	○	○

	True	False	Unsure
17. If someone has severe lung disease, that can make **general anesthesia** less safe for them.	○	○	○
18. If someone take blood thinners, that can make **general anesthesia** less safe for them.	○	○	○

After looking at the information, if you were asked to make a choice about anesthesia

SURE measure of decisional conflict	Yes	No	Unsure
18. Would you feel sure about the best choice for you?	○	○	○
19. Would you know the benefits and risks of each option?	○	○	○
20. Would you feel clear about which benefits and risks matter most to you?	○	○	○
21. Would you have enough support and advice to make a choice?	○	○	○

22. Which of the following is hot?

Fire

Ice cream

Ice cube

Snow

If your doctor showed you the grid that we shared about anesthesia choices for surgery for a broken hip, how would you feel about using it to talk about your choices?

**Acceptability**	Completely disagree	Disagree	Neither agree nor disagree	Agree	Completely agree
23. I would approve of my doctor using the grid about anesthesia choices.	①	②	③	④	⑤
24. Having my doctor use the grid about anesthesia choices is appealing to me.	①	②	③	④	⑤
25. I would like my doctor to use the grid about anesthesia choices with me.	①	②	③	④	⑤
26. I would welcome my doctor using the grid about anesthesia choices.	①	②	③	④	⑤

27. Would the grid about anesthesia choices make your treatment decision (*check one*)
- o easier
- o more difficult
- o unsure

28. Do you think the grid about anesthesia choices has enough information to help someone decide on treatment options?
- o Yes
- o No

Comments:

29. Would the grid about anesthesia choices help you talk about your choice with your doctor or other people on your care team?
- o Yes
- o No
- o Unsure

Now we would like to ask a few questions about you so we know who completed this survey.

30. How old are you?
[Numerical]

31. What is your marital status? Mark only one.
- o Married

- Living as married or living with a romantic partner
- Divorced
- Widowed
- Separated
- Single, never been married

32. What gender do you identify with?

- Female
- Male
- Non-binary
- Prefer to self-describe _________________

33. What is the highest level of formal education you have completed?

- Less than a high school degree
- A high school diploma or GED
- Technical training or certification
- Some college
- A college degree
- A graduate or professional degree

34. Are you Latino/a/x or Hispanic?

- Yes
- No

35. Which category best describes you? Mark all answers that apply.

- Asian
- Black or African American
- Caucasian or White
- Native American or Alaskan Native
- Native Hawaiian or other Pacific Islander
- Other, please specify__________________

36. Including yourself, how many people live in your household?

- [Numerical]

37. Which category best describes your household income last year?

- Less than $15,000
- At least $15,000 but less than $30,000
- At least $30,000 but less than $45,000
- At least $45,000 but less than $60,000
- At least $60,000 but less than $75,000
- $75,000 or more
- Prefer not to answer

Thank you for answering our survey questions. You may close out of the survey.
